# Note on the Use of Antitoxin

**Published:** 1896-09

**Authors:** A. Dagenais

**Affiliations:** Buffalo, N.Y. 473 Virginia Street


### NOTE ON THE USE OF ANTITOXIN.

By DR. A. DAGENAIS, Buffalo, N. Y.

ON A RECENT visit to Paris the opportunity was offered me by Dr. E. Roux, successor of Pasteur, to visit l'Hopital des Enfants Malades, where Dr. Roux's serum (antitoxin) is in constant use, as it may be required in cases in this hospital. I there met Dr. Petit, assistant of Dr. Sylvestre, who has charge of the diphtheritic patients, who kindly pointed out certain details with reference to the administration of antitoxin that I communicate to the readers of the Journal, trusting they will be of considerable interest to them, as they were to me.

Two quarantine buildings have been erected, separated from the main structure of the hospital, which is a very large institution. These separate buildings are only one story high, clean to perfection, well lighted and ventilated. Two rooms are reserved for the incoming patients that are brought in from a few hours to a few days after infection.

They are first given an injection, after which the results are constantly watched by both physicians and nurses. No other remedies are used. There is a constant flow of steam from boiling water, creating a moist atmosphere in the room, a simple mouth wash of carbonate of soda, and that is all.

The quantity of serum injected is from 10 to 20 cm. ; sometimes 50 cm. are given, but this is very seldom. When they receive at the hospital neglected cases, a second injection is occasionally needed by such, but this is never done under twenty-four hours after the first dose is injected. The injections are made over the abdomen. Odunt's tubes are generally used, or Sylvestre's, which are a modification of Odunt's.

As a matter of fact, the results of the use of the serum, according to careful observations, have simply reduced a most dreaded disease to one of comparative freedom from direful results. The simplicity of this management impressed me deeply, and its successfulness cannot be challenged.

473 Virginia Street.

---

Simple Diarrhea.—The indications are (*Canadian Practitioner*) to first remove by purgatives the irritating and decomposing contents of the intestines. This is best done by giving calomel in small doses, say one-tenth of a grain, frequently repeated, or by a full dose of castor oil.

The second indication is to withhold all food which would be likely to undergo fermentation and add to the existing toxemia. Milk and other foods should be absolutely prohibited. The child should be allowed to take pure water quite freely. Barley water, to which a little white of egg or sugar has been added, may be given, and later whey may also be given.

Third. If ptomaines are thought to be present in the lower bowel, it would be well to irrigate after each movement of the bowels, using a warm normal salt solution (1 dram. to one quart), about one pint at a time.

Finally, such drugs as retard fermentation, *e. g.*, bismuth subnit., grs. x., every two or three hours; or soda benzoate in 4-grain doses in water every two hours.—J. Lewis Smith in *Pediatrics*, July, 1896.

